# Burden of diabetes mellitus in Weifang: Changing trends in prevalence and deaths from 2010 to 2021

**DOI:** 10.1371/journal.pone.0312871

**Published:** 2024-10-30

**Authors:** Xiaoyang Wang, Danyang Ye, Min Chen, Liwen Song, Jiaxiang Bian, Lingyu Huang, Lixia Cheng

**Affiliations:** 1 School of Clinical Medicine, Shandong Second Medical University, Weifang, China; 2 Department of Prevention and Treatment of Chronic Non-communicable Diseases, Weifang Center for Disease Control and Prevention, Weifang, China; 3 Department of Endocrinology, Weifang People’s Hospital, The First Clinical Hospital of Shandong Second Medical University, Weifang, China; 4 Department of Critical Care Medicine, Fujian Medical University Union Hospital, Fuzhou, China; 5 Department of Clinical Laboratory, Weifang People’s Hospital, Weifang, China; Lithuanian University of Health Sciences, LITHUANIA

## Abstract

**Objective:**

The objective of this study is to analyze the death characteristics and burden of disease (BOD) in diabetes mellitus (DM) patients in Weifang from 2010 to 2021. The findings will serve as a foundational data source and theoretical framework for public health administrative departments in the formulation of DM-related policies.

**Methods:**

The annual percent change (APC) and average annual percent change (AAPC) of the disability-adjusted life years (DALY), years of life lost (YLL), and years lived with disability (YLD) in DM residents from 2010 to 2021 were analyzed using the Joinpoint software to reflect the changing trend of the BOD in DM patients. Additionally, we conducted an analysis of the various causes of death among these patients and compared BOD in diabetic patients with different backgrounds.

**Results:**

From 2010 to 2021, the burden of disease, which includes DALY, YL, and YLD, has been increasing among patients with DM in Weifang. It is noteworthy that the burden of disease is particularly pronounced among male patients and those aged 75 or above. Additionally, it is observed that widowed and illiterate DM patients have comparatively longer survival times. Furthermore, among the DM patients who have unfortunately passed away, it has been identified that unspecified DM with ketoacidosis accounts for 10.03% of the deaths as a direct cause of death. In contrast, type 2 diabetes mellitus (T2DM) with kidney complications contributes to 10.23% of the deaths as the fundamental cause of death.

**Conclusion:**

The city is faced with a significant challenge of diabetes, which is influenced by factors such as gender, age, cultural background, and marital status. Unspecified diabetes mellitus (DM) with ketoacidosis (10.03%) and T2DM with renal complications (0.23%) are identified as the primary direct and underlying causes of death among diabetic patients, respectively. This study serves as a valuable reference for other regions in terms of diabetes prevention, control, and the management of chronic diseases.

## Introduction

Diabetes mellitus (DM) has emerged as a prevalent chronic disease worldwide. Its incidence continues to rise steadily, posing a significant threat to public health. Without the implementation of effective mitigation measures, DM is projected to escalate to become a leading cause of death and disability globally. A recent study reported that the global count of DM patients in 2021 reached 529 million, with a corresponding age-standardized prevalence rate of 6.1%. These figures highlight the urgency to address the escalating burden of DM, as it is estimated that by 2050, the number of affected individuals will exceed a staggering 1.31 billion [1]. The number of DM patients in China is also rising. Based on the national cross-sectional representative survey data published in JAMA (the *Journal of the American Medical Association*) on 170,287 adults (2013–2014) and 173,642 adults (2018–2019) in the Chinese mainland, the estimated prevalence of DM increased from 10.9% in 2013 to 12.4% in 2018 [2]. According to the IDF Diabetes Atlas 2021 (10th Edition), China, India, and Pakistan have the highest number of adult DM patients aged 20 to 79 in 2021. This trend is projected to continue until 2045. Moreover, the high mortality rates and risk factors associated with DM are a major concern. In 2019, DM was the direct cause of 1.5 million deaths, with 48% of these deaths occurring before the age of 70. Additionally, DM was responsible for 460,000 deaths due to kidney diseases. Furthermore, elevated blood glucose levels may contribute to approximately 20% of deaths from cardiovascular diseases. The diabetes mortality rate among individuals under 25 years of age in low-income countries is approximately five times higher than that in high-income countries [3].

Regularly updating the risk assessment of BOD in patients with DM can offer prompt and precise information for the development and evaluation of preventive and treatment strategies for DM. Although previous studies have reported the global or national BOD, there is currently no scientific assessment of the BOD in residents with DM in Weifang. Many articles have analyzed the BOD in DM patients using public databases [4–6]. The objective of this study is to analyze the trends of the direct cause of death, fundamental cause of death, and disability-adjusted life years (DALY) in patients with DM in Weifang from 2010 to 2021. Additionally, we aim to evaluate the survival time of these DM patients with different sociodemographic backgrounds. The insights gained from this research will be particularly valuable in enhancing population health and life expectancy in the future.

## Materials and methods

Weifang City, located in the central part of the Shandong Peninsula, holds a strategic position at the heart of the peninsula city cluster. With an expansive area of 16,100 square kilometers, Weifang boasts a combination of distinctive marine and continental climate characteristics. Its geographical advantages have established it as a significant hub for the economy, culture, and transportation in Shandong province. As per the findings of the seventh national census, the city’s permanent resident population reaches an impressive figure of 9,386,705, ranking it as the third most populous city in the province.

### Data source

Our research focused on analyzing 9,187 fatalities related to diabetes that occurred among the residents of Weifang. We collected the data on these deaths from the Cause of Death Registration Reporting Information System.

### Data collection

At all levels, it is the responsibility of medical institutions to complete and submit death certificates and report cards. The county (city, district) level Centers for Disease Control and Prevention (CDCs) are tasked with collecting, reviewing, and ensuring the quality of death data for residents within their jurisdiction. The collected data encompassed various demographic information such as age, ethnicity, occupation, education level, marital status, and the time from onset to death for patients. Furthermore, the CDCs are also responsible for timely communication with departments of maternal and child health, departments of civil affairs, and other relevant departments. This is done in order to compare data from the maternal and child health system with the cremation data from Civil Affairs Bureaus. Such comparisons help identify and rectify any missing reports. In terms of disease classification, professionals determine the disease and cause of death codes based on the guidelines provided by ICD-10 [7] and ICD-O-3.

### Ethical approval

This study was approved by the Ethics Committee of Weifang Municipal Center for Disease Control and Prevention, the need for informed consent was waived by the committee.

### Study population and time period

The study focused on deceased patients diagnosed with diabetes in Weifang City. All residents in Weifang were included as subjects for the study. The data utilized in the research comprised survey data and health system data collected from 2010 to 2021. A total of 9187 individuals were included. The age of DM patients in this study ranged from 15 to 107 years old.

### Identification of DM and causes of death

The diagnostic code ICD-10 was used to identify DM. The death certificates of the patients provided both the fundamental and direct causes of death. Each death certificate documented the full causal chain of the patient’s disease progression.

### Calculation of DALY

Based on DALY, the calculation of BOD was performed. DALY consists of two components: years of life lost (YLL) and years lost due to disability (YLD) [6]. YLL is calculated based on incidence, while YLD is calculated based on prevalence [8, 9]. The calculation of YLD based on prevalence offers several advantages compared to the method based on incidence. One major advantage is that it does not require the knowledge of disease duration. Additionally, in Weifang, Shandong Province, China, the age-standardized DALY, YLL, and YLD were calculated based on the standard population proportion. This approach ensures comparability and accuracy in evaluating the burden of the disease.

The YLL, YLD, and DALY of residents in Weifang were calculated based on the methodologies of GBD and WHO (World Health Organization) using the following formulas [10, 11].


$$YLL_{ij}=N_{ij}\times L_{ij}$$

The age group and gender are represented by i and j respectively in the formula. N represents the number of patients who died from a certain cause in a specific age group and gender, while L represents YLL in a specific age group, which corresponds to the age point of death in the standard life table. This study utilized the standard life expectancy table provided by GBD 2019.


$$YLD_{Study\;Area\;ij}=\frac{YLD_{Reference\;Districts\;ij}}{YLL_{Reference\;Districts\;ij}}\times YLL_{Study\;Area\;ij}$$

In Weifang, the distribution of YLL from 2010 to 2021 exhibited similarities with the national distribution published by GBD (r = -0.1407632, p = 0.4129). Consequently, China was chosen as the reference area. Since the YLD-YLL ratio for China in 2021 had not been published by GBD, the estimation of YLD in Weifang was derived from the reference value in 2019.


$$DALY_{ij}=YLL_{ij}+YLDi_{j}$$

Based on the population data from the sixth national population census in China, the age standardization rate has been calculated.

Standard Life Expectancy Table of GBD.

### Statistical analysis

To estimate the long-term trend of mortality attributable to DM, a Joinpoint regression analysis was conducted due to the non-linear trend observed in cross-time BOD analysis. Joinpoint regression, also referred to as change point regression, was initially introduced by Kim et al. [12].

To assess the magnitude of mortality trends in individuals with diabetes mellitus (DM), we utilized Joinpoint regression analysis to estimate the annual percent change (APC), average annual percent change (AAPC), and their corresponding 95% confidence intervals (CIs). The definitions of APC and AAPC are as follows:

$$AAPC=\left(e^{\frac{\sum {w_{i}\beta }_{i}}{\sum w_{i}}}-1\right)*100\%$$

$$APC=\left[\frac{Y_{X}+1-Y_{X}}{Y_{X}}\right]*100\%=(e^{\beta_{1}}-1)*100\%$$

The Joinpoint model analysis was conducted using the Joinpoint software (V5.0).

## Results

**Fig 1:**
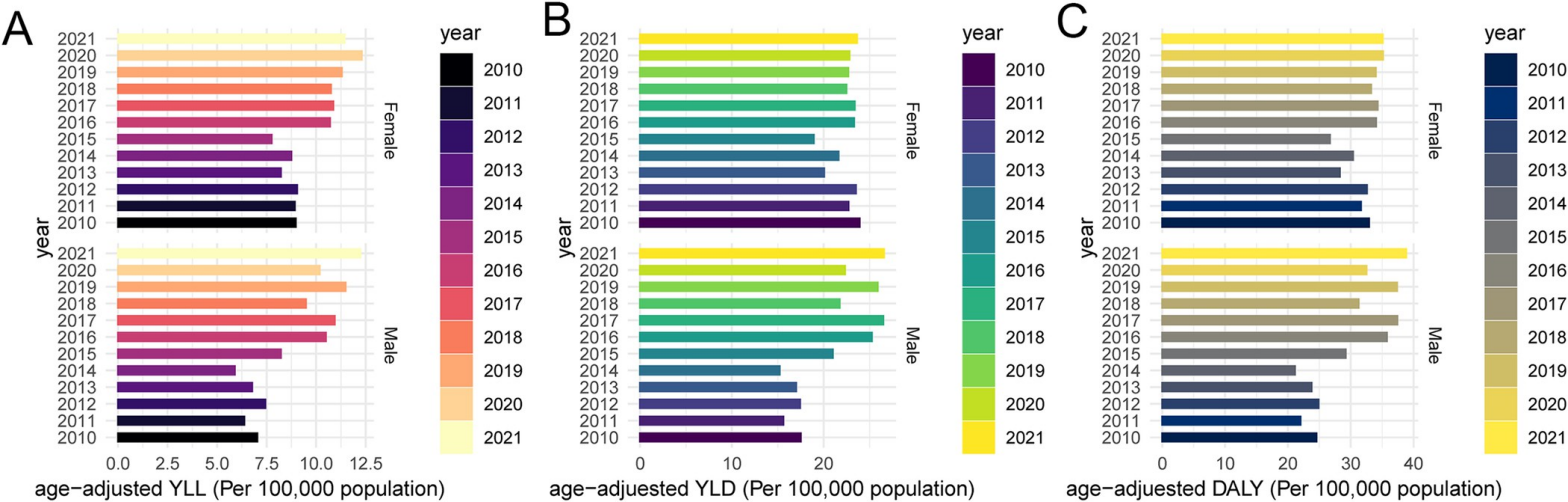
Histogram of BOD. A, B, and C show the YLL, YLD, and DALY in DM patients with different gender from 2010 to 2021.

The YLL of patients with diabetes by sex from 2010 to 2021 is illustrated in Fig 1A. Likewise, Fig 1B presents the YLD in patients with diabetes by sex during the same period. Moreover, Fig 1C showcases the DALY in patients with diabetes by sex over the aforementioned 12-year span. It is evident that there has been a substantial rise in the disease burden among individuals of both genders who have diabetes.

**Fig 2:**
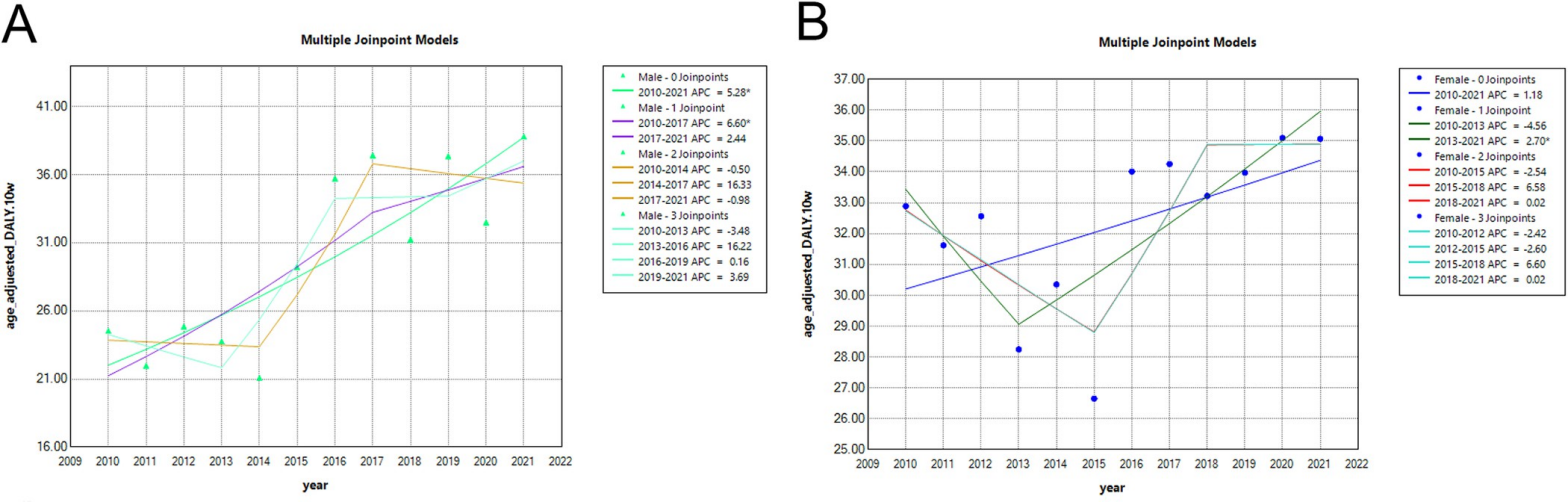
Joinpoint regression. A shows the high mortality in DM patients in Weifang from 2010 to 2021, B shows the high DALY in DM patients in Weifang from 2010 to 2021.

Fig 2A depicts the significant increase in mortality among patients with DM in Weifang from 2010 to 2021. Notably, there was a steady rise in the APC of the DALY among male patients during this period (APC = 5.28*). Specifically, from 2010 to 2017, the APC for male DM patients experienced a significant upward trend (APC = 6.60*). However, from 2017 to 2021, although there was a rising trend in the APC for male DM patients, the change was not statistically significant (APC = 2.44).

Fig 2B illustrates the considerable burden of DALY in patients with DM in Weifang from 2010 to 2021. It is evident that the overall trend of APC in DALY among female DM patients in Weifang has been increasing but without statistical significance (APC = 1.18). During the period from 2010 to 2013, there was a downward trend in the APC of DALY among female DM patients, albeit not statistically significant (APC = -4.56). However, from 2013 to 2021, there was a significant upward trend in the APC of DALY among female DM patients (APC = 2.7*).

As shown in **S1 Table**, the DALY in male patients in Weifang from 2010 to 2017 increased significantly, with an APC of 6.6008* and an AAPC of 5.0703. From 2017 to 2021, the rising change of the DALY in male patients was not significant, with an APC of 2.4445 and an AAPC of 3.6518.

According to the data presented in **S2 Table**, the decline in DALY among female patients in Weifang from 2010 to 2013 was not statistically significant, with APC of -4.5589 and AAPC of 0.6649. However, from 2013 to 2021, there was a significant increase in DALY among female patients, with an APC of 2.6967 and an AAPC of 3.6518.

**Fig 3:**
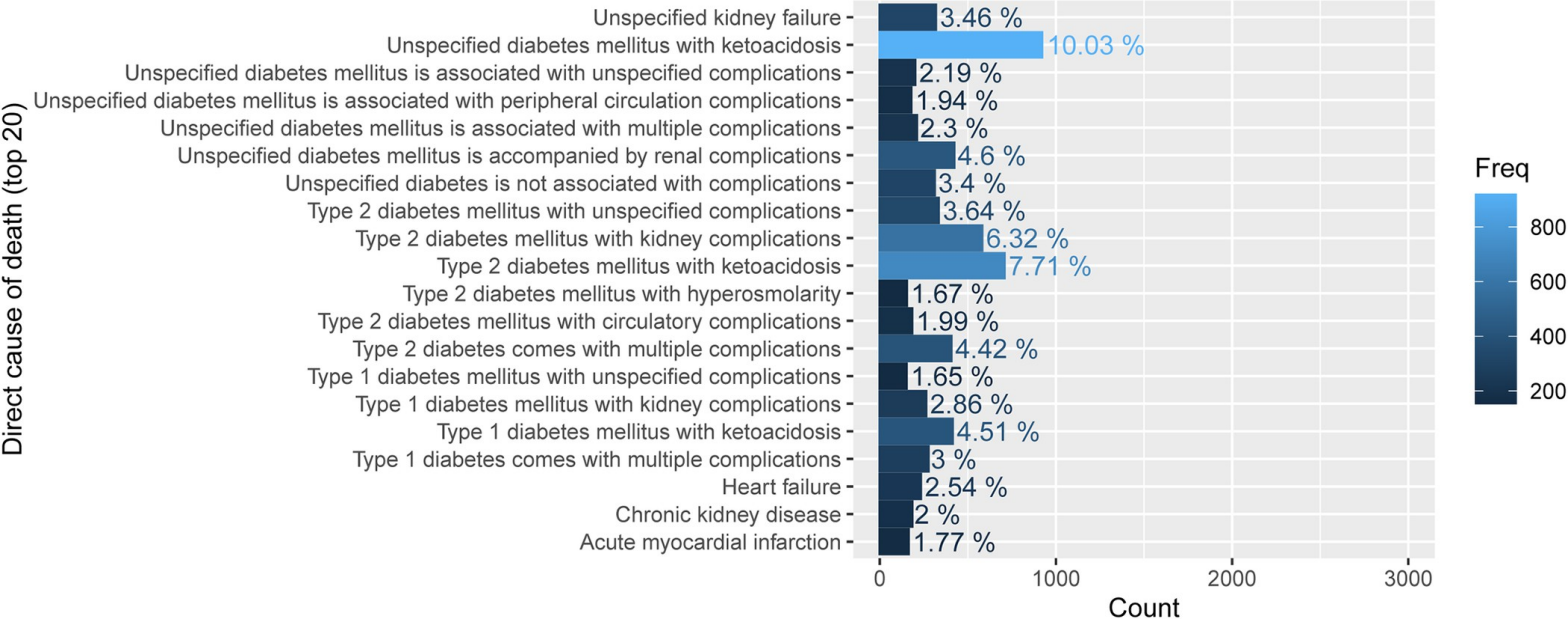
Description of direct cause of death.

According to the information presented in Fig 3, it can be observed that among the patients with DM who passed away due to the direct cause of death, the highest percentage of deaths, accounting for 10.03% (maximum), was attributed to unspecified DM with ketoacidosis. Additionally, type 2 diabetes mellitus (T2DM) with ketoacidosis accounted for 7.71% and T2DM with hypertonic hyperglycemia accounted for 1.67% (minimum) of the deaths.

**Fig 4:**
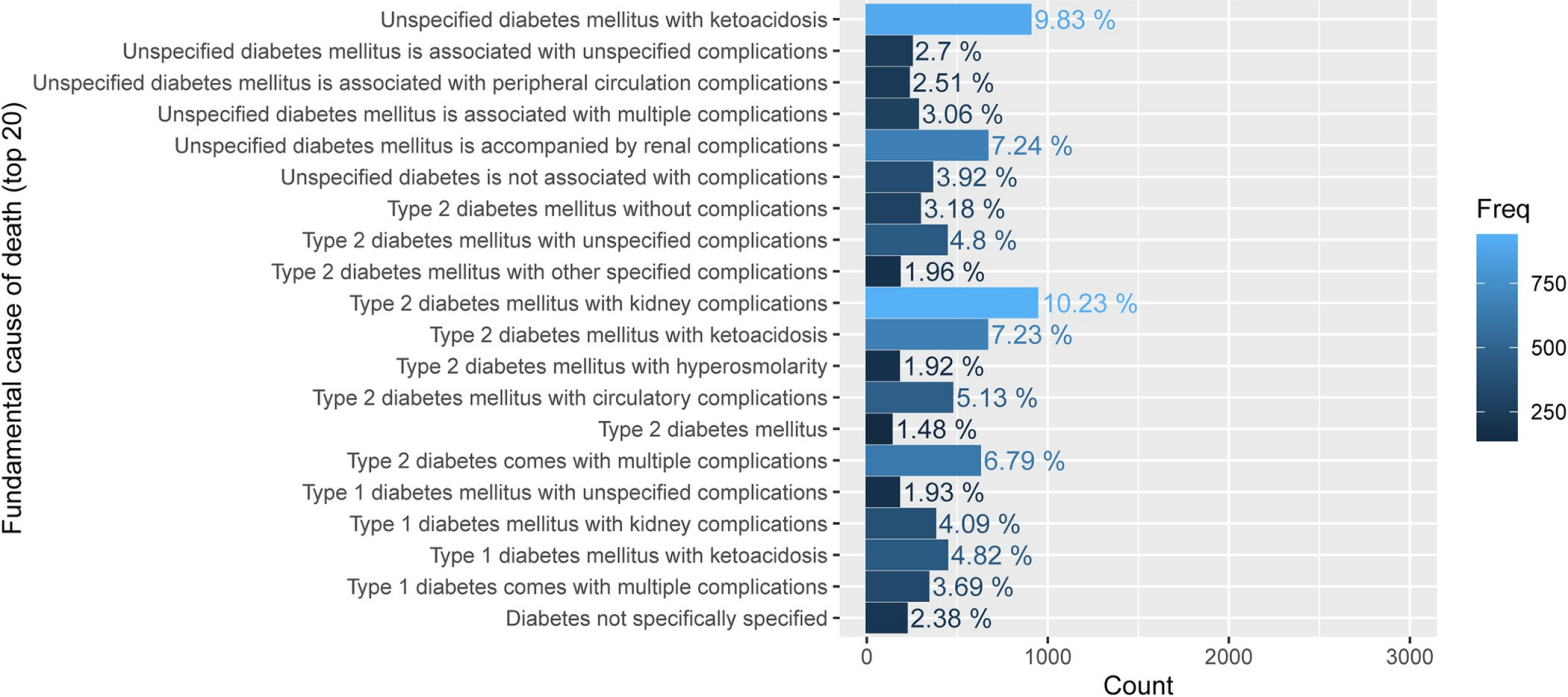
Description of fundamental cause of death.

According to Fig 4, the primary cause of death among diabetic patients was identified. T2DM with renal complications accounted for the highest percentage of deaths at 10.23. Unspecified DM with ketoacidosis was responsible for 9.83% of deaths, while T2DM with hypertonic hyperglycemia had the lowest impact at 1.92%.

**Fig 5:**
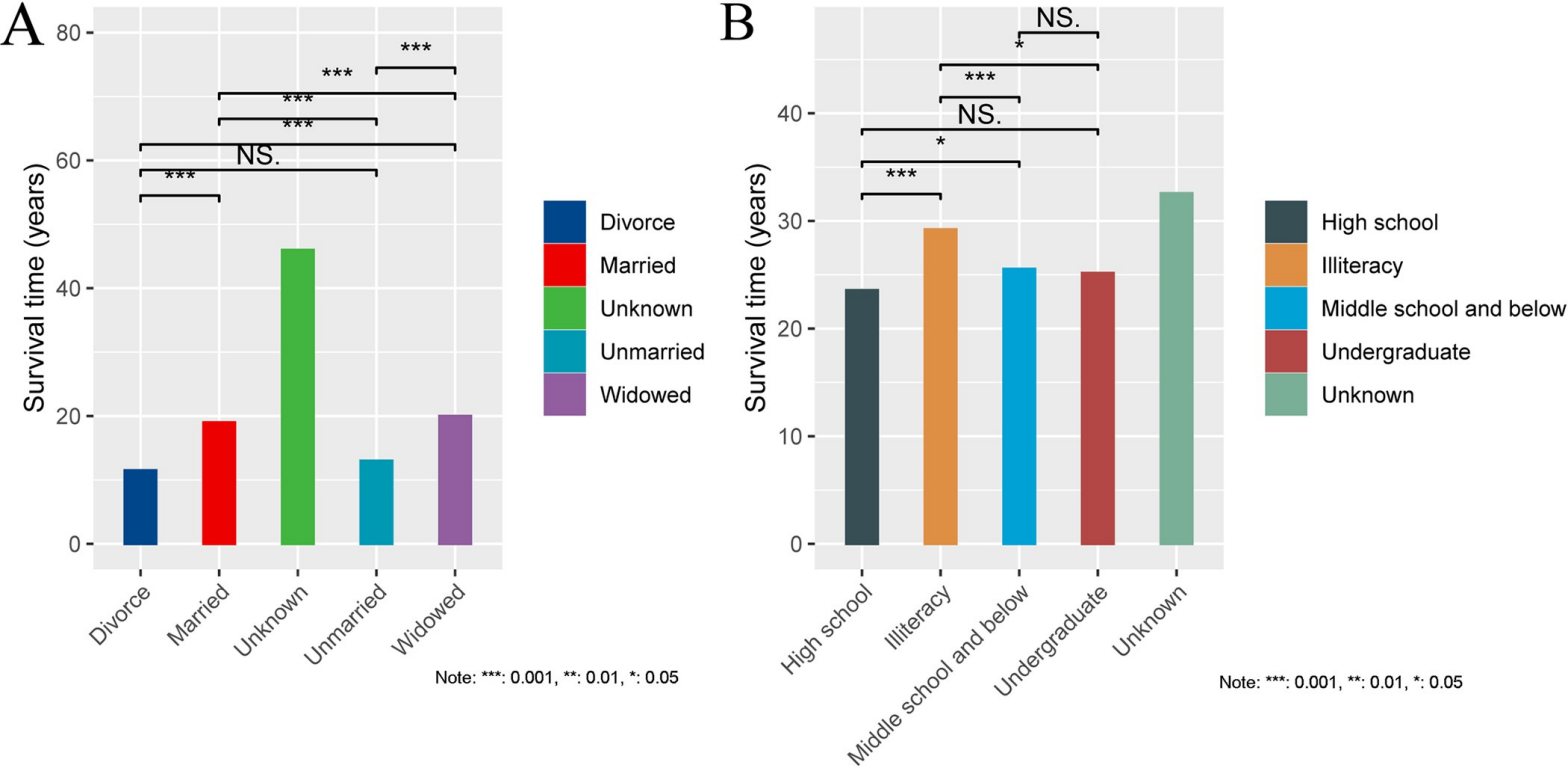
Survival time of patients with different backgrounds. A, Marital status; B, Degree of education.

According to the findings presented in Fig 5A of the study, a comparison was made among DM patients with different marital statuses regarding their survival time. The results showed that widowed patients had a significantly longer survival time compared to divorced patients (P = 3e-06, indicating statistical significance). Furthermore, it was observed that married patients had a significantly longer survival time than unmarried patients (P = 5.7e-13). The survival time of widowed patients was also found to be significantly longer than that of both married patients (P = 4.3e-14) and unmarried patients (P < 2.22e-16). On the other hand, married patients demonstrated a significantly longer survival time than divorced patients (P = 0.00037). Additionally, the study did not find a significant change in the survival time of unmarried and divorced patients (P = 0.8, indicating no statistical significance). The statistical significance level is denoted as *** for 0.001, ** for 0.01, and * for 0.05.

Among DM patients with different levels of education, we observed significant differences in survival time as illustrated in Fig 5B. Specifically, illiterate patients had a significantly longer survival time compared to patients with a high school education background (P = 1.3e-11), middle school or lower education (P = 1.4e-13), and undergraduate or higher education (P = 0.028). Patients with a middle school or lower education background also had a significantly longer survival time than those with a high school education background (P = 0.012). However, we found no statistical difference in survival time between patients with a middle school or lower education background and those with an undergraduate or higher education background (P = 0.95). Additionally, there was no significant change in survival time between patients with an undergraduate or higher education background and those with a high school education background, and the comparison result was not statistically significant (P = 0.32). (***: p < 0.001, **: p < 0.01, *: p < 0.05)

**Fig 6:**
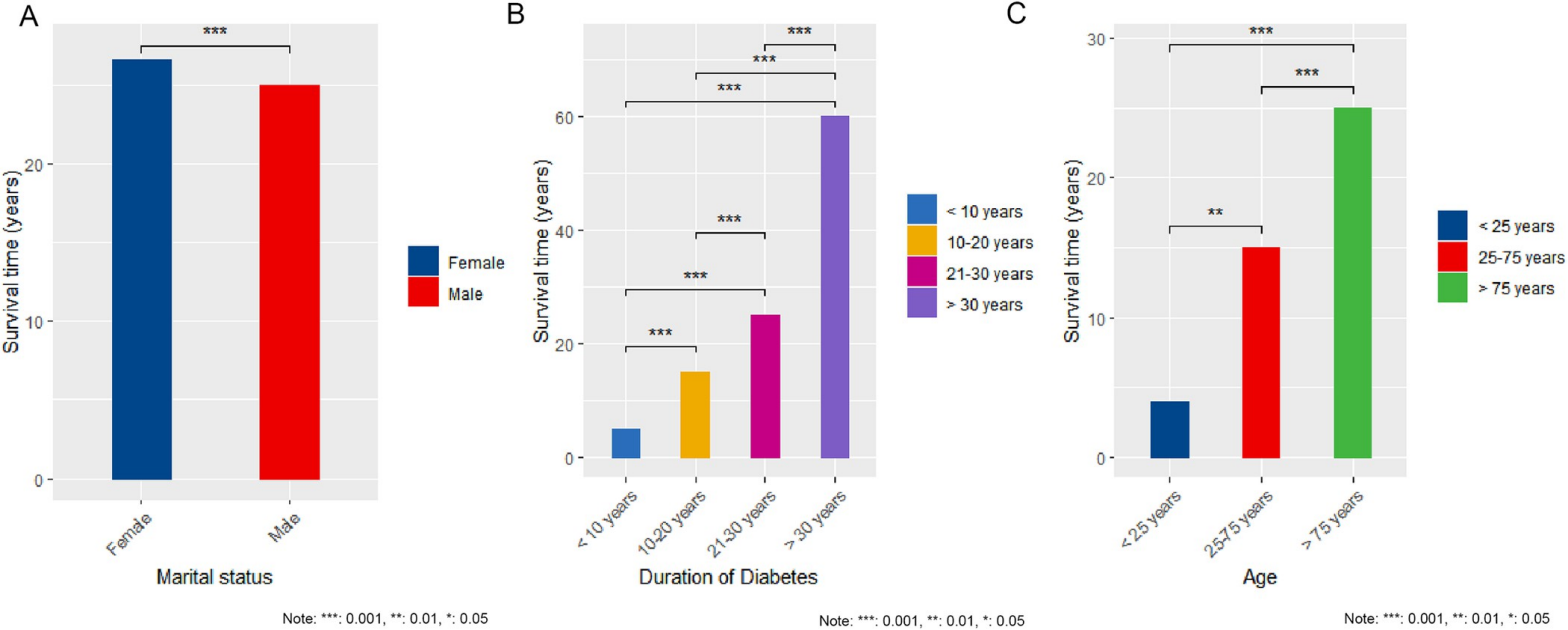
Survival time of patients with different gender age and course of disease. A, Gender status; B, Duration of diabetes; C, Age status.

As in Fig 6A, the survival time of female DM patients was significantly longer than that of male DM patients (P = 1.3e-07). (***: 0.001, **: 0.01, *: 0.05)

As shown in Fig 6B, the patients were grouped according to the course of diabetes. The results revealed that the course of diabetes was proportional to the survival time. (***: 0.001, **: 0.01, *: 0.05).

Fig 6C depicts the survival time of patients with DM (diabetes mellitus) across various age groups. To facilitate comparison, the patients were categorized into four age groups: < 25 years old, 25–49 years old, 50–74 years old, and ≥ 75 years old. Notably, a considerable number of deaths were recorded. Our analysis revealed several significant findings. First, the survival time of DM patients < 25 years old was significantly shorter compared to patients aged ≥ 75 years old (P = 0.028). Second, DM patients aged 25 to 49 exhibited significantly shorter survival times in comparison to those aged 50 to 74 (P < 2.22e-16). Additionally, the survival time of DM patients < 25 years old was significantly shorter than that of DM patients aged 50 to 74 years old (P = 0.0041). Interestingly, DM patients aged ≥ 75 years old displayed significantly longer survival times than both DM patients aged 25 to 49 (P < 2.22e-16) and DM patients aged 50 to 74 (P < 2.22e-16). However, we did not observe a significant difference in survival time between DM patients < 25 years old and those aged 25 to 49 (P = 0.2). The statistical significance of the findings is denoted by asterisks, where *** represents a significance level of 0.001, ** represents 0.01, and * represents 0.05.

## Discussion

We conducted a Joinpoint regression analysis to analyze the BOD in patients with DM in Weifang, which was one of the strengths of our study. Furthermore, we provided the first description and analysis of the causes of death in DM patients. Unlike previous studies that focused on the global or national level, our study specifically investigated a specific city, adding a unique and specific perspective. Additionally, the data collection process in our study was rigorous and systematic, with trained personnel following standardized protocols over a period of 10 years.

The worldwide prevalence of DM is on the rise, leading to an increase in the number of patients who will experience complications. It is projected that managing DM and its associated complications will pose a significant challenge for developing nations over the next 25 years. This study highlights that diabetic complications are among the top ten leading causes of death in DM patients. Specifically, DM patients with kidney complications and unspecified DM with ketoacidosis rank first in terms of mortality. Diabetic kidney disease (DKD) and kidney injury attributed to DM are reported to be the most prevalent complications, affecting approximately 40% of DM patients [13]. The potential cause could be that the aforementioned complications do not show any symptoms. By the time they are accurately diagnosed, the conditions have already reached a critical stage. Once DKD advances into end-stage renal disease (ESRD), the patient will need renal replacement therapy, which can result in significant mortality rates and impose a substantial social and economic burden [14]. Our findings align with the conclusions drawn in the study conducted by JaeJin AN et al. It was reported in their study that the high level of BOD in patients with DM is associated with the presence of at least one complication related to DM [15]. According to the research conducted by Karine Chevreul et al., it has been found that DM is a leading cause of blindness, amputation, and the need for dialysis among adult patients. Furthermore, cardiovascular disease has been identified as the primary cause of death in individuals with DM. While France has made significant progress in mitigating the economic burden associated with DM through comprehensive health insurance coverage, there is still a need for enhancing preventive measures, monitoring strategies, and intervention methods to reduce the occurrence of complications [16]. According to the research conducted by Nish Chaturvedi, it is believed that the growing number of patients with T2DM will result in an escalation in the rates of morbidity and mortality related to cardiovascular diseases over time [17]. According to Sylvia Kehlenbrink et al., there is still a lack of understanding in the scientific literature regarding the challenges faced by patients with DM in low- and middle-income countries within the humanitarian context. These patients often encounter significant hurdles in accessing medical services due to the exorbitant costs involved. In order to fully comprehend the epidemiological aspects of DM in such environments, further research is imperative [18].

DM on individuals’ quality of life is substantial and can result in a significant number of complications and premature deaths. Concerns have arisen regarding the fact that over one-third of deaths related to DM occur in individuals who are below the age of 60 [19]. A cross-sectional study was conducted by Sajjad Haider et al. on a total of 180,824 patients with diabetes mellitus (DM) who were 12 years old or older. The study utilized the IMRD database. The findings of the study indicated that the prevalence rate of DM and diabetic retinopathy (DR) has been increasing steadily from 1998 to 2018. Additionally, it was observed that the proportion of patients with type 1 diabetes mellitus (TIDM) who experience visual impairment or visual loss was higher [20]. JinChi Xie et al. conducted an evaluation of BOD in patients with T2DM aged 15 to 39 between 1990 and 2019. Their findings revealed that the age-standardized DALY in adolescents and young adults aged 15 to 39 exhibited an increasing pattern. Additionally, they noted that young women under the age of 30 were particularly susceptible to developing T2DM [21]. Moin Uddin Ahmed et al. conducted a study involving 27,931 patients with DM who were at least 45 years old. The findings revealed that BOD was more pronounced in male patients and in patients who were at least 65 years old [9]. Our study investigated the attribution of DALY in most patients to the loss of life years or fatal burden caused by lower limb amputation related to DM. We examined patients from all age groups who died from DM in Weifang. The results indicated that male patients and those aged ≥ 75 years had a more prominent burden of disease. This finding contrasts with the study conducted by Moin Uddin Ahmed et al., and may be due to differences in the number of patients surveyed and age groups analyzed. Furthermore, in Weifang, endocrinologists regularly carry out science popularization activities and provide free clinical consultations related to DM. These efforts aim to enhance DM patients’ understanding of the disease itself and discourage behaviors such as soaking or rubbing their feet in hot water. Immediate hospitalization is ensured for patients who notice foot ulcers or infections, allowing for prompt blood glucose control. Hand and foot surgeons are also invited to participate in joint consultations, diagnoses, and treatments. These collaborative measures significantly shorten the disease course and reduce the risk of diabetic foot complications.

Our research findings indicate that there has been a consistent increase in APC, AAPC, and DALY among both male and female patients with DM from 2010 to 2021. Moreover, we found that the education level and marital status of DM patients have an influence on their survival time. Specifically, we observed that widowed DM patients had a significantly longer survival time compared to married, unmarried, and divorced patients. Additionally, illiterate DM patients had a significantly longer survival time compared to those with a middle school or lower, high school, and undergraduate education background. It is important to note that there may be other contributing factors, such as geographical limitations, that could also play a role in these differences.

Based on a statistical prediction by Moien Abdul Basith et al. [19], using data from 1990 to 2017, it is projected that the global prevalence rate of DM will increase to 7,079/100,000 by 2030 and 7,862/100,000 by 2040. Despite the late onset of T2DM, it ranks seventh among the leading causes of disability and DALY. *The Medium- and Long-term Plan for the Prevention and Treatment of Chronic Diseases in China* (2017–2025) aims to reduce premature mortality, in patients aged 30 to 70, resulting from major chronic diseases such as cardiovascular and cerebrovascular diseases, cancer, chronic respiratory diseases, and DM, by 20%25 by 2025 compared to the rates in 2015. This indicator resembles life expectancy, as it is unaffected by population composition and can be compared across different periods and regions. Although there have been limited studies on the probability of premature death from DM alone, this study analyzes the mortality characteristics and burden of disease in DM patients in Weifang from 2010 to 2021, providing vital data and a theoretical foundation for the public health authorities in Weifang to formulate policies concerning DM. This will ultimately contribute to better prevention and control of DM and the overall management of chronic diseases.

However, it is important to acknowledge the limitations of our study. Firstly, we utilized data from the GBD 2019, which may have certain shortcomings. These include limited availability of crucial data, variations in case definitions, and potential collinearity among covariates. Consequently, there is a possibility of significant deviations in the accuracy of these data. Secondly, it is crucial to exercise caution when extending the conclusions of our study beyond the specific context of Weifang. The outcomes derived from the data gathered in Weifang may not necessarily be applicable to other countries or cities within China. Thirdly, males are more likely to develop T2DM and die from this disease. This is related to the fact that in the age group with a high incidence of DM, the prevalence of diabetes is higher in men than in women [22]. Furthermore the higher incidence of DM in men may also be closely related to their lifestyle, such as long static working hours, a lack of exercise, a high intake of salt and fat, smoking, and drinking [23]. Hence, males are more likely to expose to risk factors. Fourthly, there is currently no conclusive scientific evidence to support that the survival time of widowed DM patients is significantly longer than that of other marital statuses. The reason why being widowed has a positive impact on the health of DM patients may be that widowed patients may receive more attention and support from family, friends, communities, and support groups. This positive social support may help reduce life stress and improve psychological state and enhance their life satisfaction. Fifthly, we must acknowledge that other influential factors that could impact the burden of diabetes were not extensively analyzed in our study. These factors include the number of family members the patient has, their history of diabetic complications, smoking habits, and alcohol consumption. Sixthly, it is worth noting that the majority of deceased diabetic patients included in our study were over 50 years old. This demographic is characterized by a significant proportion of illiterate individuals, as compulsory nine-year education was implemented in China in 1986. This disparity in educational background may introduce additional complexities and potential biases in our analysis. Furthermore, it is important to recognize that the analysis process was impacted by incomplete data provided by patients. This may have resulted in missing values and unclear subtypes, potentially introducing bias into our findings.

## Conclusion

From 2010 to 2021, the burden of diabetes, measured in Years of Life Lost (YLL), Years Lived with Disability (YLD), and Disability-Adjusted Life Years (DALY), has continued to rise in Weifang City. Survival time among diabetic patients is influenced by various factors, such as gender, age groups, educational backgrounds, and marital statuses. Among deceased diabetic patients, unspecified DM with ketoacidosis (10.03%) and T2DM with renal complications (10.23%) are the first direct cause of death and the first underlying cause of death, respectively. It is important to note that there is a lack of comprehensive studies analyzing the direct and fundamental causes of death in DM. Our study aims to fill this research gap and provide insights that can inform prevention and control strategies for DM, as well as the management of chronic diseases in different regions or nations.

## Supporting information

S1 Table Comparison of DALY in male DM patients in Weifang from 2010 to 2021. a. Annual Percent Change (APC), b. Average Annual Percent Change (AAPC).
(DOCX)

S2 Table Comparison of DALY in female DM patients in Weifang from 2010 to 2021. a. Annual Percent Change (APC), b. Average Annual Percent Change (AAPC).
(DOCX)
