# Supplementary material for: Burden of diabetes mellitus in Weifang: Changing trends in prevalence and deaths from 2010 to 2021
Source: PLoS One. 2024 Oct 30;19(10):e0312871. doi: 10.1371/journal.pone.0312871 (PMC11524517; doi:10.1371/journal.pone.0312871)
Supplement: S1 Table — a. Annual Percent Change (APC), b. Average Annual Percent Change (AAPC). (DOCX) [file pone.0312871.s001.docx]

# Supporting information

**S1 Table. Comparison of DALY in Male DM Patients in Weifang from 2010 to 2021.** a. Annual Percent Change (APC), b. Average Annual Percent Change (AAPC).

**a.**

| **Cohort** | **Segment** | **Lower Endpoint** | **Upper Endpoint** | **APC** | **Lower CI** | **Upper CI** | **Test Statistic (t)** | **Prob > \|t\|** |
| --- | --- | --- | --- | --- | --- | --- | --- | --- |
| Male - 0 Joinpoints | 1 | 2010 | 2021 | 5.2750* | 2.4116 | 8.263 | -- | -- |
| Male - 1 Joinpoint | 1 | 2010 | 2017 | 6.6008* | 0.207 | 13.4026 | 2.4437 | 0.044518 |
| Male - 1 Joinpoint | 2 | 2017 | 2021 | 2.4445 | -11.5046 | 18.5924 | 0.3902 | 0.708014 |
| Male - 2 Joinpoints | 1 | 2010 | 2014 | -0.4997 | -10.5184 | 10.6407 | -0.1311 | 0.902059 |
| Male - 2 Joinpoints | 2 | 2014 | 2017 | 16.3343 | -16.8318 | 62.7266 | 1.2517 | 0.278886 |
| Male - 2 Joinpoints | 3 | 2017 | 2021 | -0.978 | -10.9485 | 10.1088 | -0.2571 | 0.809766 |
| Male - 3 Joinpoints | 1 | 2010 | 2013 | -3.4804 | -58.9231 | 126.7946 | -0.5269 | 0.691294 |
| Male - 3 Joinpoints | 2 | 2013 | 2016 | 16.2161 | -78.951 | 541.6541 | 1.1176 | 0.464686 |
| Male - 3 Joinpoints | 3 | 2016 | 2019 | 0.1645 | -81.8583 | 453.0296 | 0.0122 | 0.99222 |
| Male - 3 Joinpoints | 4 | 2019 | 2021 | 3.69 | -81.2197 | 472.4948 | 0.2695 | 0.83243 |

**b.**

| **Cohort** | **Range** | **Lower Endpoint** | **Upper Endpoint** | **AAPC** | **Lower CI** | **Upper CI** | **Test Statistic~** | **P-Value~** |
| --- | --- | --- | --- | --- | --- | --- | --- | --- |
| Male - 0 Joinpoints | Full Range | 2010 | 2021 | 5.2750* | 2.4116 | 8.263 | -- | -- |
| Male - 1 Joinpoint | Full Range | 2010 | 2021 | 5.0703 | -0.5396 | 10.9966 | 1.7667 | 0.07728 |
| Male - 2 Joinpoints | Full Range | 2010 | 2021 | 3.6518 | -3.8595 | 11.75 | 0.9345 | 0.35005 |
| Male - 3 Joinpoints | Full Range | 2010 | 2021 | 3.9107 | -7.6538 | 16.9234 | 0.6372 | 0.523966 |
