# Supplementary material for: Burden of diabetes mellitus in Weifang: Changing trends in prevalence and deaths from 2010 to 2021
Source: PLoS One. 2024 Oct 30;19(10):e0312871. doi: 10.1371/journal.pone.0312871 (PMC11524517; doi:10.1371/journal.pone.0312871)
Supplement: S2 Table — a. Annual Percent Change (APC), b. Average Annual Percent Change (AAPC). (DOCX) [file pone.0312871.s002.docx]

# Supporting information

**S2 Table.** **Comparison of DALY in Female DM Patients in Weifang from 2010 to 2021.** a. Annual Percent Change (APC), b. Average Annual Percent Change (AAPC).

**a.**

| **Cohort** | **Segment** | **Lower Endpoint** | **Upper Endpoint** | **APC** | **Lower CI** | **Upper CI** | **Test Statistic (t)** | **Prob > \|t\|** |
| --- | --- | --- | --- | --- | --- | --- | --- | --- |
| Female - 0 Joinpoints | 1 | 2010 | 2021 | 1.182 | -0.3111 | 2.7299 | -- | -- |
| Female - 1 Joinpoint | 1 | 2010 | 2013 | -4.5589 | -14.5274 | 6.5722 | -1.0002 | 0.350524 |
| Female - 1 Joinpoint | 2 | 2013 | 2021 | 2.6967* | 0.2541 | 5.1988 | 2.6139 | 0.034717 |
| Female - 2 Joinpoints | 1 | 2010 | 2015 | -2.5374 | -6.3023 | 1.3788 | -1.8113 | 0.144321 |
| Female - 2 Joinpoints | 2 | 2015 | 2018 | 6.5772 | -10.6387 | 27.1097 | 1.0038 | 0.372256 |
| Female - 2 Joinpoints | 3 | 2018 | 2021 | 0.0213 | -8.4127 | 9.232 | 0.0067 | 0.994967 |
| Female - 3 Joinpoints | 1 | 2010 | 2012 | -2.4222 | -23.731 | 24.8399 | -1.2645 | 0.425964 |
| Female - 3 Joinpoints | 2 | 2012 | 2015 | -2.5991 | -23.8692 | 24.6137 | -1.3581 | 0.40406 |
| Female - 3 Joinpoints | 3 | 2015 | 2018 | 6.6045 | -16.6755 | 36.3886 | 3.2982 | 0.18741 |
| Female - 3 Joinpoints | 4 | 2018 | 2021 | 0.0153 | -11.577 | 13.1273 | 0.0157 | 0.989976 |

**b.**

| **Cohort** | **Range** | **Lower Endpoint** | **Upper Endpoint** | **AAPC** | **Lower CI** | **Upper CI** | **Test Statistic~** | **P-Value~** |
| --- | --- | --- | --- | --- | --- | --- | --- | --- |
| Female - 0 Joinpoints | Full Range | 2010 | 2021 | 1.182 | -0.3111 | 2.7299 | -- | -- |
| Female - 1 Joinpoint | Full Range | 2010 | 2021 | 0.6649 | -2.198 | 3.6115 | 0.4502 | 0.652585 |
| Female - 2 Joinpoints | Full Range | 2010 | 2021 | 0.5765 | -3.3647 | 4.6784 | 0.2818 | 0.778072 |
| Female - 3 Joinpoints | Full Range | 2010 | 2021 | 0.5861 | -1.1108 | 2.3121 | 0.6732 | 0.50084 |
